# Why Does the Halophyte *Mesembryanthemum crystallinum* Better Tolerate Ni Toxicity than *Brassica juncea*: Implication of Antioxidant Defense Systems

**DOI:** 10.3390/plants9030312

**Published:** 2020-03-02

**Authors:** Taoufik Amari, Aymen Souid, Rim Ghabriche, Mauro Porrini, Stanley Lutts, Gian Attilio Sacchi, Chedly Abdelly, Tahar Ghnaya

**Affiliations:** 1 Laboratoire des PlantesExtrêmophiles, Centre de Biotechnologie de Borj-Cédria, BP 901, Hammam-lif 2050, Tunisia; taoufik.amari@gmail.com (T.A.); souid_aymen2008@yahoo.fr (A.S.); Rimghabriche@gmail.com (R.G.); chedly.abdelly@gmail.com (C.A.); 2 Department of Agricultural and Environmental Sciences, UniversitàdegliStudi di Milano, 20133 Milan, Italy; Mauro.Porrini@unimi.it (M.P.); gianattilo.sacchi@unimi.it (G.A.S.); 3 Groupe de Recherche enPhysiologieVégétale (GRPV), Earth and Life Institute, Université Catholique de Louvain, 1348 Louvain-la-Neuve, Belgium; stanley.lutts@uclouvain.be; 4 Higher Institute of Arts and Crafts of Tataouine, University of Gabes Erriadh City, Zrig-Gabes 6072, Tunisia

**Keywords:** antioxidant systems, glycophyte, halophyte, nickel stress, phytoremediation

## Abstract

The implication of enzymatic and non-enzymatic antioxidative systems in response to Ni was evaluated in the halophyte *Mesembryanthemum crystallinum* in comparison with the metal tolerant glycophyte species *Brassica juncea*. Seedlings of both species were hydroponically subjected during 21 days to 0, 25, 50, and 100 µM NiCl_2_. Growth parameters showed that the halophyte *M. crystallinum* was more tolerant to Ni than *B. juncea*. Malondialdehyde (MDA) content increased to a higher extent in *B. juncea* than in *M. crystallinum*. Antioxidant enzymesactivities were differently affected by Ni in both species. Nickel increased shoot superoxide dismutase (SOD) and ascorbate peroxidase (APX) activities in *B. juncea*, whereas these activities were reduced in *M. crystallinum* when exposed to metal stress. The root SOD, APX and guaiacol peroxidase (GPX) activities increased upon Ni treatments for both species. The content of non-enzymatic antioxidative molecules such as glutathione, non-protein thiols and proline increased in Ni-treated plants, except for GSH content in the shoot of *B. juncea.* Based on the oxidative balance, our findings confirm the higher tolerance of the halophyte *M. crystallinum* to Ni-induced oxidative stress comparatively to *B. juncea.* We suggest that *M. crystallinum* is able to overcome the produced ROS using the non-enzymatic system, while Ni-induced oxidative stress was more acute in *B. juncea*, leading this species to mainly use the enzymatic system to protect against reactive oxygen species.

## 1. Introduction

Anthropogenic activities including industrialization, urbanization and agricultural practices are the main factors responsible for an excessive accumulation of toxic metals in the environment. These non-biodegradable elements accumulate in the superficial soil horizons, groundwater and surface water. They may then be absorbed by plants and constitute a major risk for human contamination by the trophic chain [1,2,3]. These pollutants are indeed frequently reported as the primary causes of several human pathologies [4]. Their extraction from contaminated soils thus constitutes a priority for ecosystem stability and human health. In this context, several physicochemical methods have been proposed, but most of them are very expensive, require sophisticated equipment and specialized staff, and may destroy soil texture and telluric microorganisms. Nickel (Ni) is one of the most potentially harmful heavy metals for living organisms [5,6]. It has been released into the environment through various sources such as smelting, industrial wastes, burning of fossil fuel, mining and organic manures [6,7]. In plants, it is well documented that Ni is an essential micronutrient and plant cannot complete their life cycle without adequate levels of this metal (0.01 to 10 mg g^−1^ DW) [8]. Nickel is indeed required for normal activities of several enzymes (metalloenzymes), including urease involved in nitrogen metabolism of higher plants [9]. However, an excess of Ni becomes toxic for most plant species [10].

In plants, Ni is essentially absorbed through the root system via both passive diffusion and active transport [7] (Yusuf et al., 2011). However, the relative involvement of each pathway in total Ni uptake by plants varies between species as well as the chemical speciation and concentration of Ni in soil or nutrient solution [7,11]. The molecular basis of Ni absorption is still not well understood, although many transporters from the ZRT/IRT-like (ZIP) family are thought to be involved in this process [12]. De la Torre et al. [13] suggested that IRT1 is the essential route for Ni uptake in *A. thaliana*, since under Fe limiting conditions, the wild-type is able to accumulate 3 times more Ni than *irt1* mutant. An increased Ni uptake was also reported in *Arabidopsis tahliana* under Zn-deficiency suggesting the implication of Zn-transporter ZIP3 in Ni absorption [11]. However, in the Ni-hyperaccumulators *Alyssum inflatum* and *Alyssum bracteatum*, Ni up take is not affected by Zn concentrations in the medium [14]. Some members of Nramp (natural resistance-associated macrophage protein) family were also reported to be directly involved in Ni transport in plants [15]. 

Excess Ni induces severe physiological/biochemical alterations and leads to symptoms of toxicity, such as chlorosis and foliar necrosis, ultimately culminating in a sharp decline in crop production [8]. Nickel may impair photosynthesis, water use efficiency, mineral uptake, and thus results in growth retardation [16,17,18]. Numerous studies reported thatNi phytotoxicity induces oxidative stress due to reactive oxygen species (ROS) overproduction [19] such as hydroxyl radicals (OH·), superoxide anion (O_2_**^−^**) and hydrogen peroxide (H_2_O_2_) [10,16,20]. As a non-redox-active metal, Ni^2+^ cannot directly generate these oxidative molecules, but it may indirectly interfere with the antioxidant system responses. High levels of reactive oxygen species (ROS) in plant tissues can oxidize biological macromolecules, leading to major cellular damages, such as DNA alterations, oxidation of proteins and lipid peroxidation [21,22,23]. 

Some plant species are nevertheless able to cope with elevated Ni content in their shoots without expressing these toxicity symptoms. These so-called “hyperaccumulating plant species”, Develop a wide range of defense mechanisms involving enzymatic and non-enzymatic antioxidant systems, which can alleviate cellular oxidative damages by scavenging ROS species.Gill and Tuteja [23]; Ghnaya et al. [24]; Mnasri et al. [25] and Amari et al. [26] demonstrated that, under heavy metals stress, some halophyte plant species (*Sesuvium portula-castrum* and *Mesembryanthemum crystallinum*) are able to fix metals to organic and amino acids. It was demonstrated that, in plants exposed to abiotic stresses, free proline exhibits a powerful antioxidant activity required to avoid ROS deleterious effects [19,23,27]. This amino acid could be also directly involved in metal ion chelation [28,29,30]. Glutathione (GSH) metabolism could also play an important role in maintaining the oxidative status in plants exposed to metal stress [31]. GSH is an essential tripeptide (γ-glutamylcysteinyl glycine, γ-Glu–Cys–Gly) that plays a fundamental dual role: first, as an antioxidant to mitigate the redox imbalance caused by toxic metal accumulation and second, as a precursor of the ligand peptides phytochelatins responsible for free metal ion chelation and compartmentation in the vacuole [32]. Furthermore, several key antioxidants enzymes, such as catalase (CAT), guaïacol peroxidase (GPX), superoxide dismutase (SOD) and the enzymes of the ascorbate glutathione cycle including ascorbate peroxidase (APX) assume key functions in the ROS detoxifying process [33,34] and it was shown that the activities of these major antioxidant enzymes can be significantly enhanced in plants subjected to Ni toxicity [35]. 

In numerous areas of the world, salt affected areas with low population often constitute sites for industrial and urban wastes contaminated with heavy metals [36,37]. Most of the Ni-hyperaccumulating plants are glycophytes species unable to tolerate salinity and consequently cannot be used to extract metals from salty contaminated soils. More recently, it has been demonstrated that halophytes, are able to cope with metal constraint more efficiently than glycophytes [24,38,39,40], suggesting their interest for phytoextraction of heavy metals on salt-affected substrates. 

In an earlier study, we demonstrated that the halophyte *Mesembryanthemum crystallinum* accumulated much more Ni^2+^, than the glycophyte *Brassica juncea* (glycophyte) [18,26]. Interestingly, the halophyte species showed a better aptitude to maintain photosynthetic activity and pigment concentrations and to preserve the PSII functional integrity when challenged with Ni excess [18]. The ability of *M. crystallinum* to tolerate high Ni concentration could be related to the accumulation of organic acids and histidine in tissues, which are potential metal ligands that specifically bind nickel in the cytosol or in subcellular compartments [26]. The role of the antioxidant defense system in Ni^2+^-tolerance of *M. crystallinum* remains poorly studied and the involvement of enzymes and non-enzymatic compounds in plants exposed to toxic doses of Ni^2+^ still needs to be clarify. Therefore, the aim of the present study was to perform a comparative evaluation of the antioxidant defense in *M. crystallinum* (halophyte) and *B. juncea* (glycophyte) when subjected to nickel stress, through physiological parameters related to lipid peroxidation and enzymatic/non-enzymatic antioxidant system. 

## 2. Materials and Methods

### 2.1. Plant Material and Culture Conditions

The seeds of *Mesembryanthemum crystallinum* were collected from their natural habitat near Thina (Sfax, 300 km South of Tunis) while those of *Brassica juncea* (Acc PI 173874) were kindly provided by the North Central Regional Plant Introduction Station (NCRPIS-USDA-USA). Seeds of both species were sterilized using 10% H_2_O_2_ solution during 20 min, then washed with distilled water and sown on perlite imbibed with distilled water placed for 4 days in the dark at 24 ± 1 °C room temperature and 70% relative humidity. Two weeks after germination, plants were grown hydroponically for 3 weeks in pots filled with 5 L continuously aerated Hoagland’s nutrient solution (8 plants per pot) [41]. The experiment was carried out under growth chamber conditions with 25 ± 1/18 ± 1 °C temperature, 55/75% relative humidity (RH) and16 h/8 h photoperiod day/night regime. Solutions were renewed every 3 days to avoid depletion of nutrients and Ni concentrations. pH was adjusted to 5.8. An initial harvest was performed just before Ni application to characterize the initial plant size and weight. Ni treatments were applied to obtain a final concentration of 0, 25, 50 and 100 µM NiCl_2_ (Sigma-Aldrich) (3 pots per treatment). After 3 weeks, Ni-treated and control plants were harvested and used for physiological and biochemical analyses.

### 2.2. Growth Analysis

At the harvest, plants (6 replicates per treatment) were divided into shoots and roots. Shoots were rinsed three times with cold distilled water and blotted between two layers of filter paper. Roots were immediately dipped in a cold solution of HCl (0.01 M) for 5 min to dissolve metals adsorbed on the root surface [42] and then washed three times with cold distilled water and blotted with filter paper. The fresh weight was immediately determined, and the dry weight was measured after 48 h of desiccation in an oven at 60 °C.

The relative growth rate (RGR) was calculated according to Hunt (1990) [43]:

RGR = lnM_2_-LnM_1_/(t_2_ − t_1_)

where M_2_ and M_1_ were the dry matter at the final and initial harvests, respectively, and (t_2_ − t_1_) was the nickel treatment duration (days).

### 2.3. Estimation of Lipid Peroxidation

The extent of lipid peroxidation was estimated by determining the amount of malondialdehyde (MDA) in plant tissues by the method of Hodges et al. [44], which takes into account the possible influence of interfering compounds in the thiobarbituric acid (TBA)-reactive substances assay. MDA contents were measured on the roots and leaves of *B. juncea* and *M. crystallinum* exposed to all treatments. Fresh samples were extracted for 45 min with 80:20 (*v/v*) ethanol/water using an ultrasonic cleaner (Bransonic, Danbury, CT). The homogenate was centrifuged at 15,000× *g* for 10 min. Then the pellet was reextracted twice with the same solvent. Supernatants were pooled and an aliquot was added to a test tube with an equal volume of either (1) − TBA solution containing 20% (*w/v*) trichloroacetic acid and 0.01% (*w/v*) BHT, or (2) + TBA solution containing the above added with 0.65% (*w/v*) TBA. Samples were heated at 95 °C for 25 min and after cooling, absorbance was read at 440, 532 and 600 nm. MDA equivalents were calculated as 106 × [(A − B)/157,000], where A = (Abs 532 + TBA) − (Abs 600 + TBA) − [(Abs 532−TBA) − (Abs 600 − TBA)], and B = (Abs 440 + TBA) − [(Abs 600 + TBA) × 0.0571].

### 2.4. Enzyme Extractions

All of the following operations were performed at 4 °C. The fresh leaves and roots samples (0.5 g) were rapidly extracted in a prechilled mortar with 10% (*w/w*) PVP (polyvinylpyrrolidone) in 50 mM K-phosphate buffer (pH 8), containing 0.1 mM EDTA (ethylenediaminetetraacetic acid), 1 mM DTT (dithiothreitol), and 0.5 mM PMSF (phenyl-methyl-sulphonyl-fluoride). For APX activity, 20 mM ascorbate was added to the extraction medium to maintain the enzyme active during extraction. The homogenates were centrifuged at 12.000× *g* for 30 min. For APX, the supernatant was dialyzed for 2 h against the same buffer used for the homogenization containing 5 mM sodium ascorbate. Three replicates per treatment were used. The supernatants were collected and their protein concentrations were determined according to Bradford [45], using bovine serum albumin as a standard.

### 2.5. Enzyme Assays

The total activity of the SOD (EC 1.11.1.5) in the roots and shoots was measured according to Scebba et al. [46]. Different volumes of organ crude extracts (5, 10, 20, and 40 µL) were added to the reaction mixture reaching a final volume of 3 mL. The reaction mixture contains 50 mM potassium phosphate buffer (pH 7.8), 0.1 mM EDTA, 13 mM L-methionine, 2 µM riboflavin and 75 µM NBT (nitroblue tetrazolium). The reaction was started by exposing the mixture to cool white fluorescent light for 15 min. The blue reaction color was measured spectrophotometrically at 560 nm. The control reaction mixture had no enzyme extract, while blanks had the same complete reaction mixture, but were kept in the dark. The volume of sample that induces 50% inhibition of color development was considered as one unit of SOD activity and the activity was expressed as units per mg of protein.

The total activity of CAT (EC 1.11.1.6) was assayed spectrophotometrically referring to the method of Luck [47] by monitoring the decline in absorbance at 240 nm as H_2_O_2_ was consumed, against a plant extract-free blank. The 3 mL reaction mixture contained 66 mM sodium phosphate buffer (pH 7.0), to which 30% (*w/v*) H_2_O_2_ was added (the optical density was around 0.5 at 240 nm with a 1 cm light path). The reaction was initiated by adding an appropriate dilution of the shoot or root crude extract to this solution. The time required for a decrease in the optical density of from 0.45 to 0.4 was used for CAT activity calculations. CAT activity was expressed as unit per mg of protein. A unit is the amount of an enzyme which liberates half the peroxide oxygen from a hydrogen peroxide solution of any concentration in 100 s at 25 °C.

The total APX (EC 1.11.1.11) activity was measured according to Nakano and Asada [48] by following the decline in absorbance at 290 nm as ascorbate was oxidized (R = 2.8 mM^−1^ cm^−1^). The rate of ascorbate oxidation was estimated between 1st and 60th s after starting the reaction with the addition of H_2_O_2_. The 1 mL reaction mixture contained 50 mM HEPES-NaOH (pH 7.6), 0.22 mM ascorbate, 1 mM H_2_O_2_ and an enzyme sample. The control reaction mixture was prepared without adding the enzyme extract. Corrections were made for the low, non-enzymatic oxidation of ascorbate by H_2_O_2_ and for the oxidation of ascorbate in the absence of H_2_O_2_. The activity was expressed as units (µmol of oxidized ascorbate per min) per mg of protein.

The total GPX activity was determined according to Fielding and Hall [49] by following the increase in absorbance at 470 nm when adding the enzymatic preparation to 2 mL of guaiacol (0.5%) and 9 mM hydrogen peroxide (H_2_O_2_) in K–phosphate buffer (50 mM, pH 7.0).

### 2.6. Non-Protein Thiols Determination

NPT concentration was determined according to Rijstenbil et al. [50]. Briefly, 100 mg of freeze-dried tissue of roots and leaves were crushed and homogenized in ice. The powder was suspended in 1 mL of a mixture of 236 mM sulfosalicylic acid SSA, 6.3 mM diethylenetriamine pentaacetic acid (DTPA) at pH 2. This solution was used to keep the thiols in reduced state. Homogenates were sonicated. Cell residues were removed by centrifugation at 12,000× *g* for 20 min at 8 °C. The SH group of non-protein thiol forms a DTNB (dithionitrobenzoic acid)-SH complex that was spectrophotometrically measured at 412 nm. In each case, at least 5 samples were analyzed.

### 2.7. Glutathione Determination

The aliquots of the fresh leaf and root tissue (0.5 g) were crushed and homogenized in ice-cold 5% (*w/v*) TCA, using a cold mortar and pestle, then centrifuged at 15,000× *g* for 10 min at 4 °C. Total (GSH + GSSG) and oxidized glutathione (GSSG) were determined in the supernatant by the 5,5-dithiobis-nitrobenzoic acid (DTNB)-GR recycling procedure as reported in Sgherri and Navari-Izzo [51]. Changes in the absorbance of the reaction mixture were measured at 412 nm at 25 °C. The total glutathione concentration was calculated from a standard curve in which GSH equivalents (1–10 nmol) were plotted against the rate of change at 412 nm.

### 2.8. Free Proline Concentration

For proline determination, the frozen root and leaves samples were homogenized in 3 mL of 1 mM tridecafluoroheptanoic acid (TDFHA), 50% (*v/v*) methanol. Samples were shaken for 10 min at 4 °C and then centrifuged twice at 14,000× *g* for 20 min at 4 °C. The underivatized supernatant was finally diluted to 0.5 mM TDFHA, 25% (*v/v*) methanol. The LC-ESI-MS analysis was conducted using an Agilent Technologies 1200 Series capillary pump coupled with dual ESI source on a 6520 Q-TOF mass spectrometer according to Armstrong et al. [52]. Briefly, LC runs were done on an XDB-C18 column (2.1 × 50 mm, 1.8 μm, Agilent Technologies) applying a 30 min non-linear gradient of 0.5 mM TDFHA/acetonitrile with a flow rate of 200 μL/min. The ESI source was set at 350 °C, 3500 V, and fragmentor at 100 V. The data acquisition range was 50–350 m/z at 0.93 scans/s. The quantization was conducted on EIC for single MH^+^ in a ± 0.02 m/z window, accepting a mass error of ± 5 mDa in ion identification and referring to calibration curves in the adequate concentration range.

### 2.9. Statistical Analysis

ANOVA with orthogonal contrasts and mean comparison procedures were used to detect differences between treatments. Mean separation procedures were conducted using Duncan’s multiple range tests with least significant difference (LSD) (*p* < 0.05).

## 3. Results

### 3.1. Plant Morphology and Growth 

After eight days of Ni treatment, chlorosis was visible in young leaves of *B. juncea* exposed to Ni^2+^. One week later, chlorosis intensity increased and necrosis appeared in oldest leaves, with a subsequent falling of these senescing leaves at the highest Ni^2+^ concentrations (50 and 100 μM). In contrast, such toxicity symptoms were not observed on leaves of Ni-treated *M. crystallinum* plants, even at the highest Ni concentration (100 μM). Both root (RDW) and shoot biomasses (SDW) decreased significantly in the two considered species with increasing Ni^2+^ concentrations (Table 1). 

On average, the reduction percentage observed for the whole plant biomasses production at 50 µM as compared to control reached 35% and 58% in *M. crystallinum* and *B. juncea*, respectively (Table 1). Based on this parameter, the halophyte *M. crystallinum* maintained a better growth in the presence of Ni as compared to *B. juncea*. 

*Mesembryanthemum crystallinum* was characterized by a lower RGR as compared to *B. juncea* (0.081 and 0.12 d^−1^ respectively) on Ni-free solution. The detrimental effect of Ni^2+^ exposure on the plant growth activity was more pronounced in *B. juncea* than *M.crystallinum* as reflected by the sharper decrease of RGR in the former species (−55% as compared to the control values at 50 µM NiCl_2_ versus −21% in *M. crystallinum*).

### 3.2. Ni Effect on Malondialdehyde (MDA) and Proline Tissus Contents

**Figure 1:**
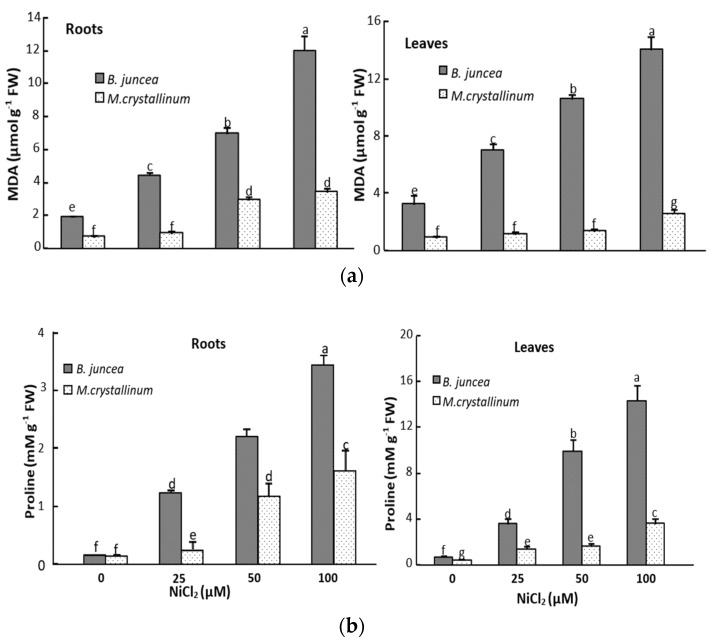
MDA (**a**) and proline (**b**) concentrations in the roots and leaves of *M. crystallinum* and *B. juncea* subjected during 21 d to different Ni concentrations. According to ANOVA, LSD 0.05 test, means (*n* = 6 per treatment ± SE) marked with the same letters are not significantly different.

The determination of the oxidation of polyunsaturated fatty acids estimated by the production of the MDA showed increased values in leaves of Ni-treated *B. juncea* (Figure 1) as compared to their respective controls. For instance, compared to the control, the MDA content increased by 260% at 100 µM NiCl_2_ in *B. juncea* whereas it was only slightly affected in *M. crystallinum* leaves whatever the Ni dose (Figure 1a). The lipid peroxidation was significantly increased in the roots of both *B. juncea* and *M. crystallinum* plants (Figure 1a). 

Ni^2+^ led to a marked increase of proline concentration in *B.juncea* leaves, reaching 600% for all Ni treatments as compared to control. In *M. crystallinum*, we also noticed a significant increase in proline concentration with increasing NiCl_2_ concentrations in the medium (Figure 1b). A similar trend was observed in the roots of both species, with a lower extent as compared to leaves (Figure 1b) and it was especially marked at the highest Ni concentration (100 µM NiCl_2_).

### 3.3. Leaf and Root Antioxidant Enzyme Activities

**Figure 2:**
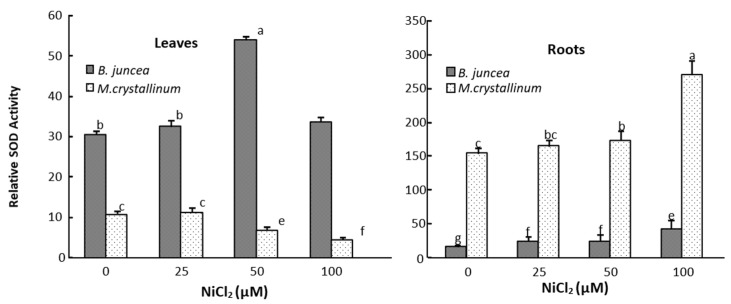
Superoxide dismutase (SOD) activity in the roots and leaves of *M. crystallinum* and *B. juncea* treated with different doses of NiCl_2_ during 21 days. According to ANOVA, LSD 0.05 test, means (*n* = 6 per treatment ± SE) marked with the same letters are not significantly different.

**Figure 3:**
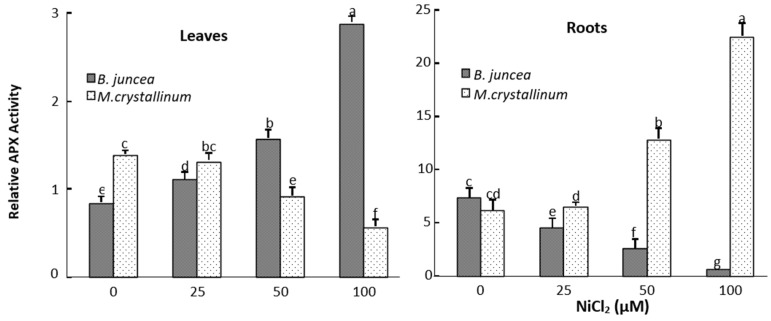
Ascorbate peroxidase (APX) activity in the roots and leaves treated of NiCl_2_-treated *M. crystallinum* and *B. juncea*. According to ANOVA, LSD 0.05 test, means (*n* = 6 per treatment ± SE) marked with the same letters are not significantly different.

**Figure 4:**
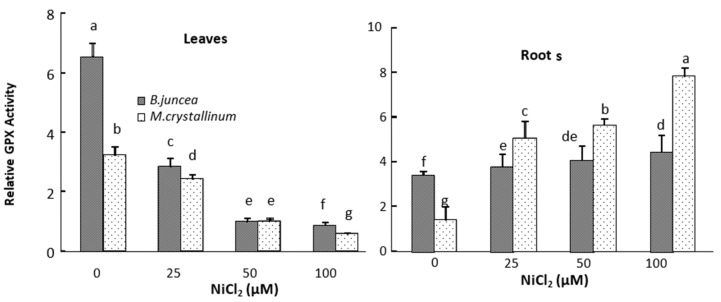
Guaiacol peroxidise (GPX) activity in the roots and leaves of *M. crystallinum* and *B. juncea* treated with NiCl_2_ during 21 days. According to ANOVA, LSD 0.05 test, means (*n* = 6 per treatment ± SE) marked with the same letters are not significantly different.

In the leaves of *B. juncea*, SOD and APX activities significantly increased under all Ni-treatments (Figure 2 and Figure 3), whereas the GPX activities decreased in Ni treated plants as compared to control (Figure 4). In contrast, SOD, APX and GPX activities significantly decreased under Ni treatments in the shoot of *M. crystallinum* shoots (Figure 2, Figure 3 and Figure 4). In the roots of both species, Ni stimulated the activities of SOD and GPX (Figure 2 and Figure 4), but the APX activity decreased in *B. juncea* roots under Ni treatment. The activity of all enzymes was higher in roots compared to leaves for both species when exposed to Ni^2+^. It is also worth mentioning that CAT show low activity in both species and was not affected by the Ni treatments.

### 3.4. Non-Protein Thiol and Glutathione Contents

Non-protein thiol (NPT) and glutathione levels variations in the root and the shoot of both species subjected to Ni are displayed in Table 2. For both species, an increase in the Ni external concentration induced an increase in the root NPT concentrations which was significant for doses higher than 25 µM. As far as the shoots were concerned, we demonstrated that 25 μM Ni did not induce a significant modification of the concentration of NPT whereas a significant increase in NPT was recorded in both species at 100 μM Ni, although it was more marked in *M. crystallinum* than in *B. juncea*. At the intermediate dose (50 µM) an increase of NPT content was recorded in the shoots of *M. crystallinum* shoots while a significant decrease in this parameter was detected in *B. juncea*. 

Nickel induced a significant increase in root GSH concentration in *B. juncea* but decreased it in the shoot. In *M. crystallinum*, the increasing Ni external concentration induced a significant proportional increase in GSH contents in both shoots and roots.

## 4. Discussion

Nickel is an indispensable micronutrient for plants growth and it is required at lower concentrations in agricultural soils [8,53]. However, an excess of this element induces toxicity and drastically reduces plant growth and productivity [7,22]. Interestingly, at the physiological and metabolomic levels, plants respond differently to the stress induced by the excess of Ni [26,54]. As we show in the present study, nickel adversely affected the plant growth in both species (Table 1). Based on plant growth parameters (DW and RGR), the halophyte *M. crystallinum* is more tolerant to Ni constraint than *B. juncea*. This tolerance was also evident when comparing plant morphology since Ni induced acute chlorosis and necrosis only in *B.juncea* plants.

Nickel is able to interfere with several other elements in several biological and chemical reactions. To date, several lines of evidence indicate that Ni toxicity in plants is associated with oxidative stress induction [10,19,55,56] as reflected by the generation of hydroxyl radicals, superoxide anions, nitric oxide and hydrogen peroxide [19,20]. The high production of reactive oxygen species (ROS) during stress disturbs the cellular redox homeostasis, by enhancing oxidative processes such as protein oxidation, enzyme activity inhibition, membrane lipid peroxidation, and DNA and RNA damage [57]. Malondialdehyde (MDA), one of the decomposition products of polyunsaturated fatty acids of membrane, is regarded as a reliable indicator of oxidative stress [58]. In our conditions, the MDA concentrations increased in both roots and leaves of Ni-treated *B.juncea* plants, indicating that an oxidative stress appeared quickly following Ni-exposure. By contrast, the low content of MDA in Ni-treated *M. crystallinum* plants compared to *B.juncea*, indicates the high aptitude of this halophyte to preserve its membrane integrity against the peroxidation induced by Ni. These data confirm that the growth of the halophyte was less affected by Ni.

Several previous data demonstrated that Ni induced MDA accumulation in many plant species. Boominathan and Doran, [55], Gajewska and Sklodowska [22,59] and Dubey and Pandey [60] reported an increase in MDA concentration in *Alyssum bertolonii*, *Triticum aestivum* and *Vigna mungo* plants when subjected to Ni, respectively. This is notably due to the destruction of cell membranes flowing attack induced by the ROS generated by Ni. In fact, chloroplasts have a complex system of membranes rich in polyunsaturated fatty acids, which are potential targets of ROS for peroxidation [61].

The oxidative degradation of lipid of chloroplast membrane under heavy metal stress provokes alterations of the thylakoids structure, thus leading to the disruption of the plant photosynthetic activity [62]. Moreover, the changes in the lipid composition and the degree of unsaturated fatty acids induced by metals can directly cause biomembrane deterioration, especially affecting the fluidity and selective permeability of the membrane [63]. Hence, in our previous investigations, we showed a significant drop in *B. juncea* nutrients acquisition and photosynthesis under Ni constraint [18]. By contrast, Ni-treated *M.crystallinum* plants showed a better aptitude to maintain photosynthetic activity and pigment concentrations and to preserve the PSII functional integrity when challenged with Ni excess [18].

Several mechanisms could govern metal resistance in plants. Among them, the enzymatic antioxidant system is an important defense strategy co-evolved with aerobic metabolism as a response to metal-induced toxicity to counteract the ROS oxidative consequences. Indeed, antioxidant enzymes such as, superoxide dismutase (SOD), guaiacol peroxidase (GPX), ascorbate peroxidase (APX) and catalase (CAT) are notably implicated in the ROS-scavenging and contribute to regulation of cellular redox balance [33]. For example, SOD is usually considered as the first line of defense against oxidative stress [64]. By controlling the steady-state superoxide levels, SOD plays an essential protective role against cellular oxidative damage, because the superoxide ion acts as a precursor of more cytotoxic or highly reactive oxygen derivatives, such as peroxynitrite or hydroxyl radical [60]. In our experiment, nickel stress enhanced the SOD activity in the leaves of Ni-treated *B.juncea* plant, whereas it induced the decrease in this activity of this enzyme in *M. crystallinum* leaves. An increase in the SOD activity was also observed in the roots of both Ni-treated species. 

In previous work, researchers demonstrated that the impact of metal constraint on the activity of antioxidant enzymes depend on the plant species, organs sampled, metal dose, and duration of exposure. For example, exposure of *Coffea arabica* [65] and *Oryza sativa* [35] to Ni provoked a significant increase in the SOD activity. Kumar et al. [66] also reported an enhanced activity of SOD in the roots and the leaves of Ni-treated *Hordeum vulgare* plants. However, Ni-treatment of *Alyssum bestolonii*, *Nicotiana tabacum* [55] and *Hydrocharisdubia* [67] resulted in a severe depletion of SOD activity.

One of the likely mechanisms which can explain the decreasing enzyme activity is related to the interaction of Ni^2+^ with ligand groups e.g., –SH of enzymes, inhibiting enzyme activity by hiding the prosthetic groups or protein denaturation [68]. Ni^2+^ may indirectly affect proteins, particularly metalloenzymes, by disturbance in the absorption of essential minerals such as Fe, Cu, Zn or Mn [69]. It can also competitively substitute the essential elements thus affecting the enzymes activity [17].

Our results showed that exposure of *B. juncea* and *M. crystallinum* plants to Ni resulted in reductions in GPX activity at the leaf level. Ni treatment also led to a marked decrease in the APX activity in the leaves of Ni-tolerant halophyte species *M. crystallinum* leaves. By contrast, an increase in this enzyme activity was observed in *B. juncea* leaves. The increase in APX activity was also observed in *Triticum aestivum* [70], *Hordeum vulgare* [71] and *Oryza sativa* leaves [35] subjected to Ni. In our study, the increase of SOD and APX activities in the shoots of *B. juncea* suggest that this metal induces the generation of their substrates, respectively, O_2_^−^ et H_2_O_2_, while the same Ni doses did not induce the production of these oxidative molecules in the shoots of *M. crystallinum*. 

In the roots of both species, Ni enhanced the GPX activity. This suggests that Ni induces the accumulation of H_2_O_2_ in the roots of tested species. Similar results have been reported in Ni-treated *Piceaabies* [72], *Sileneparadoxa* [73] and *Pisum sativum* [74] indicating a potential defensive role of GPX against Ni-induced oxidative stress. 

Contrary to APX and GPX activities, CAT activity in the *B.juncea* and *M.crystallinum* plants did not exhibit a clear cut response to Ni exposure. This is consistent with other studies showing that CAT activity generally remained unchanged in *Crotalaria juncea* [75], *Phaseolus vulgaris* [76] and *Hordeum vulgare* [66] subjected to Ni. Although, CAT has been considered among the most important antioxidant enzymes, it seems hence forth that its importance in the detoxification of H_2_O_2_ is reduced, excepting that derived from the peroxisomal metabolism in relation to photorespiration process [77,78]. Some authors believe that peroxidases assume the major role in detoxifying harmful reactive oxygen species in plants. CAT would be a “reel” to limit excessive production of ROS [79]. However, this assumption may be wrong because, although the results are frequently contradictory in the literature, the catalase activity is often negatively affected during heavy metal stress [55,70].

In addition to antioxidant enzymes, plants also use other mechanisms to scavenge reactive oxygen species in order to limit their possible destructive impact on cells. This system, commonly named the non-enzymatic antioxidative system, comprises molecules such as ascorbic acid, non-proteins thiols, α-tocopherol, polyamines and proline that can detoxify ROS and maintain their levels at non-damaging levels [23]. Non protein thiols compounds may be found in most plant and they play a key role in the regulation of the redox equilibrium and the heavy metals detoxification [31,80]. In the present study, Ni^2+^ increased the NPT content in the roots (Table 2) and the shoots in both species. These increases were more elevated in the more Ni-tolerant species, *M. crystallinum.* This is consistent with previous studies showing the increase of NPT levels in the leaves of *Oryza sativa* and *Hordeum vulgare* [35,66].

Glutathione is a well-known antioxidant playing a prominent role in the cell defense. It is a powerful reductant and hence a very efficient scavenger of ROS [81,82]. An active detoxification mechanism developed by plants, to avoid heavy metal poisoning involves intracellular sequestration of metal ions by means of glutathione and phytochelatines [83]. In this study, the GSH concentrations increased significantly in both roots and leaves of *B. juncea*. The same tendency was observed in *M. crystallinum* roots, whereas leaves GSH concentrations decreased (Table 2). This significant accumulation of GSH, especially in the roots, suggests its possible implication in Ni (II) chelation and sequestration in these organs. In addition, several earlier studies revealed that GSH acts as a first non-enzymatic line of defense against metal toxicity by directly complexing metals [83,84]. GSH is able to bind to free metal ions forming non-toxic complexes protecting cell structure and limiting the generation of oxidative stress damage [23,85]. For instance, in several Ni-hypeaccumulators species such as *Thlaspi*, it has been reported that GSH is strongly implicated in the Ni accumulation and tolerance [31]. 

In addition to low molecular -SH rich peptides, proline has long been recognized as an important protector against several abiotic stresses [86,87,88]. Proline synthesis under stress alleviates cytoplasmic acidosis and maintains the NADP^+^/NADPH ratio at functional and metabolic levels [88]. Furthermore, proline acts as osmoprotectant and membrane stabilizer [89]. Several earlier studies have also shown that proline can acts as an efficient ROS scavenger [90,91]. Our results showed that proline concentration was greatly increased in the roots and leaves of Ni-exposed plants, and the increase was more pronounced in leaves than roots in both *B.juncea* and *M.crystallinum* (Figure 1b). Our findings agree with other studies reporting proline increase in Ni-treated *Nicotiana tabacum* [92] and *Triticum aestivum* [19]. Proline accumulates in many plant species exposed to other heavy metals such as cadmium [93,94,95]. Despite several reports on proline accumulation in plants exposed to heavy metals, there is no clear consensus on the mechanism by which proline alleviates heavy metal toxicity. However, nickel, like numerous other heavy metals, can affect the plant water balance [18]. It is plausible that proline accumulated in Ni-stressed plants may be implicated in osmoregulation. On the other hand, it has been frequently reported that proline protect proteins structures [27,96], stabilizes cell membrane [86] and reduce the excessive production of ROS [19,23,91].

## 5. Conclusions

Taken together, the results of this study show that the halophyte, *Mesembryanthemum crystallinum* was more tolerant to nickel constraint than the glycophyte *B. juncea*. In fact, under the same Ni-dose, malondialdehyde content in the glycophyte species increased to a higher extent than in the halophyte. Accordingly, the activities of antioxidant enzymes (SOD, APX and GPX) in *B. juncea* increased faster than in *M.crystallinum*, suggesting a higher level of oxidative stress induced by Ni in *B.juncea*. The coordinated increase of the enzymes activities was effective in protecting the plant from the accumulation of ROS under Ni stress. However, the shoots of *M. crystallinum*, exhibited a surprising decrease in SOD activity in Ni-treated plants as compared to control ones, while Ni accumulated to higher concentration than in *B. juncea*. Furthermore, it may be suggested that the elevated level of NPT, glutathione and proline, (non-enzymatic antioxidative system), could be, at least partially, responsible for the development of resistance against nickel stress especially in *M.crystallinum*.

## Figures and Tables

**Table 1:** Of different NiCl_2_ external concentrations on root biomass (RDW), shoot biomass (SDW), the whole plant dry weight (WPDW) and relative growth rate (RGR) of *M. crystallinum* and *B.juncea* after 21 days of treatment. According to ANOVA, LSD 0.05 test and considering each species alone and for the same column, means (*n* = 6 per treatment ± SE) with at least one same letter are not significantly different.

NiCl_2_	RDW (g)	SDW (g)	WPDW (g)	RGR (d^−1^)
*M. crystallinum*				
0	0.70 ± 0.06a	2.17 ± 0.11a	2.77 ± 0.12a	0.081 ± 0.003a
25	0.47 ± 0.02b	1.62 ± 0.04b	2.09 ± 0.03b	0.075 ± 0.004b
50	0.41 ± 0.03c	1.41 ± 0.07c	1.82 ± 0.10c	0.064 ± 0.004c
100	0.30 ± 0.02d	0.89 ± 0.04d	1.19 ± 0.08d	0.049 ± 0.002d
*B. juncea*				
0	1.19 ± 0.10a	5.22 ± 0.32a	6.41 ± 0.41a	0.121 ± 0.004a
25	0.89 ± 0.09b	3.94 ± 0.28b	4.82 ± 0.37b	0.086 ± 0.004b
50	0.61 ± 0.04c	2.13 ± 0.14c	2.76 ± 0.18c	0.054 ± 0.002c
100	0.50 ± 0.05d	1.60 ± 0.21d	2.1 ± 0.26d	0.042 ± 0.003d

**Table 2:** The effect of different NiCl_2_ external concentrations on non-thiol protein (NPT) and glutathione (GSH) content of *M. crystallinum* and *B. juncea*. According to ANOVA, LSD 0.05 test and considering each species alone and for the same parameter at the same column, means (*n* = 6 per treatment ± SE) with at least one same letter are not significantly different.

NiCl_2_ (µM)	*Brassica juncea*		*Mesembryanthemum crystallinum*	
	Root	Shoot	Root	Shoot
NPT (nmol/g FW)				
0 µM	305.29 ± 29.13c	19.7 ± 0.053b	301.90 ± 26.18b	96.29 ± 7.35c
25 µM	301.47 ± 28.12c	18.76 ± 1.34b	309.90 ± 6.13b	97.99 ± 6.72c
50 µM	457.81 ± 40.74ab	14.32 ± 1.12c	547.60 ± 24.52a	156.45 ± 12.61b
100 µM	579.82 ± 53.67a	82.74 ± 5.21a	522.28 ± 31.19a	601.30 ± 55.11a
GSH (µmol/g FW)				
0 µM	0.135 ± 0.017d	0.147 ± 0.006a	0.106 ± 0.004d	0.221 ± 0.005b
25 µM	0.123 ± 0.013cd	0.076 ± 0.008b	0.136 ± 0.001c	0.213 ± 0.041c
50 µM	0.205 ± 0.012b	0.064 ± 0.005c	0.172 ± 0.015b	0.318 ± 0.034a
100 µM	0.265 ± 0.010a	0.068 ± 0.004c	0.318 ± 0.018a	0.307 ± 0.030a

## Abbreviations

APX	Ascorbate peroxidase
CAT	Catalase
DTPA	diethylenetriamine pentaacetic acid
DTNB	dithiobis-nitrobenzoic acid
DTNB	dithionitrobenzoic acid
DTT	dithiothreitol
DW	Dry Weight
ETDA	Ethylenediaminetetraacetic acid
FW; GSH	glutathione, Fresh weight
GPX	Guaiacol peroxidase
H_2_O_2_	Hydrogen Peroxide
OH	hydroxyl radicals
MDA	Malondialdehyde
NBT	nitroblue tetrazolium
NPT	Non-protein thiols
GSSG	oxidized glutathione
PMSF	phenyl-methyl-sulphonyl-fluoride
PS	Photosystem
ROS	reactive Oxygen Species
RGR	Relative growth rate
RH	Relative humidity
RDW	root biomass
SDW	shoot biomass
SSA	sulfosalicylic acid
O_2_**^−^**	Superoxide anion
SOD	superoxide dismutase
TBA	Thiobarbituric acid
TDFHA	tridecafluoroheptanoic acid
WPDW	whole plant dry weight
